# P-540. Undesired Weight Gain and Associated Healthcare Outcomes Among People Living with HIV on Antiretroviral Therapy

**DOI:** 10.1093/ofid/ofae631.739

**Published:** 2025-01-29

**Authors:** Bekana K Tadese, Janelle Cambron-Mellott, Jean Marie Arduino, Bridget L Balkaran, Shakiba Eslamimehr, José M Zuniga

**Affiliations:** Merck & Co., Inc., Rahway, New Jersey, United States, North Wales, Pennsylvania; Oracle Life Sciences, Austin, Texas; Merck & Co., Inc., Kenilworth, NJ; Oracle Life Sciences, Austin, Texas; Oracle Life Sciences, Austin, Texas; International Association of Providers of AIDS Care (IAPAC), Washington, District of Columbia

## Abstract

**Background:**

Studies indicate that people living with HIV (PLHIV) experience weight gain associated with certain classes of antiretroviral treatment (ART). Managing overall health beyond viral suppression, including weight gain, is essential for improving PLHIV quality of life. This study characterizes undesired weight gain, weight management, and associated healthcare outcomes among PLHIV.

**Methods:**

A cross-sectional Web-based survey was fielded from February-June 2022 to PLHIV in the US aged ≥18 years, and on ART. Demographic and clinical factors, weight change, weight gain associated burden/concern and health outcomes were explored. Undesired weight gain in the past 12 months was captured. Multivariable logistic regression analyses were conducted to examine associations between undesired weight gain and factors and healthcare outcomes.

**Results:**

A total of 781 PLHIV were included; median age 41 years (range:18-76); 56% cisgender male; 52% White; 25% Black. Most (86%) had undetectable viral load; 55% were living with HIV ≥5 years. About half (52%) were either overweight or obese. A total of 230 (29%) reported experiencing weight gain in the past 12 months, of which 172 (75%) reported undesired weight gain. Among those with undesired weight gain, the median (IQR) increase in weight was 20 lbs (10, 40). PLHIV with undesired weight gain were more likely to report multimorbidity and duration of HIV infection >10 years vs 1-5 years (*p*< 0.05). Over half (54%) and 23% reported that their HIV clinicians sometimes or never/rarely, respectively, discuss their weight.

In the multivariable regression, PLHIV had higher odds of undesired weight gain in those who switched their ART regimens ≥ 3 times vs those who have not (OR:3.14; 95% CI: 1.69 -5.93), obese (OR: 3.44; 95% CI: 1.97- 6.03) or overweight (OR: 2.37; 95% CI: 1.45-3.18) vs normal baseline BMI, and lower odds of undesired weight gain in cisgender female vs cisgender male (OR:0.53; 95% CI:0.33-0.84).

**Conclusion:**

This study highlights the burden of undesired weight gain and its association with obesity, comorbidities, and switching ART regimens among PLHIV. The results underscore the need for regular weight monitoring and management approaches including careful consideration of individual risks when selecting ART regimen to avert excessive weight gain.

**Disclosures:**

**Janelle Cambron-Mellott, PhD**, Oracle Life Sciences, Oracle Corporation: Employee of Oracle Life Sciences who received funding from Merck to conduct study.|Oracle Life Sciences, Oracle Corporation: Stocks/Bonds (Public Company) **Jean Marie Arduino, MS, ScD**, Merck & Co., Inc., Rahway, NJ: Employee|Merck & Co., Inc., Rahway, NJ: Stocks/Bonds (Public Company) **Bridget L. Balkaran, MPH**, Merck: Advisor/Consultant|Merck: funding for this work|Oracle Life Sciences: Employee|Oracle Life Sciences: Ownership Interest|Oracle Life Sciences: Stocks/Bonds (Public Company)

